# Supplementary material for: Evaluating an Innovative HIV Self-Testing Service With Web-Based, Real-Time Counseling Provided by an Artificial Intelligence Chatbot (HIVST-Chatbot) in Increasing HIV Self-Testing Use Among Chinese Men Who Have Sex With Men: Protocol for a Noninferiority Randomized Controlled Trial
Source: JMIR Res Protoc. 2023 Jun 30;12:e48447. doi: 10.2196/48447 (PMC10365592; doi:10.2196/48447)
Supplement: Multimedia Appendix 1 [file resprot_v12i1e48447_app1.docx]

The questions and definition of risk level are identical to those used by facility-based HIV testing and counseling services in Hong Kong

Q1. Prior to administering the official test, it would be beneficial to preliminary assess your potential risk of HIV infection. This will allow for adequate psychological preparation prior to the test results. However, if you deem risk assessment to be unnecessary, you may opt to skip this step through the available options below.

1. I would like to conduct the risk assessment – go to Q2
2. I want to skip the risk assessment

Q2. Have you been adhering to a consistent PrEP regimen? Specifically, have you been taking one PrEP tablet daily irrespective of sexual activity or taking two pills 2 to 24 hours before each anal intercourse, followed by one tablet every 24 hours for two days after such anal intercourse?

1. Take PrEP regularly – complete the risk assessment
2. Didn’t take PrEP regularly – go to Q3
3. Never take PrEP – go to Q3

Q3. Do you engage in the sharing of non-sterile needles, syringes, or mixing tools with others for the purposes of drug injection?

1. Yes – complete the risk assessment
2. No – go to Q4

Q4. Have you consistently used condoms when you had anal sex with male partners?

1. Yes – go to Q4A
2. No – go to Q4B
3. No anal sex in the last 6 months – complete the risk assessment

Q4A.Was there any occurrence of condom damage or slippage while engaging in anal intercourse?

1. Yes – go to Q4B
2. No – complete the risk assessment

Q4B. Are you aware of the HIV status of your sexual partners?

1. All sexual partners are HIV-positive – go to Q4C
2. All sexual partners are HIV-negative – complete the risk assessment
3. Some sexual partners are HIV-positive, while some are HIV-negative – complete the risk assessment
4. No disclosure – complete the risk assessment

Q4C. Has the level of HIV in the body of sexual partners who have been infected remained undetectable for a duration of six months?

1. Yes - complete the risk assessment
2. No - complete the risk assessment
3. Uncertain - complete the risk assessment
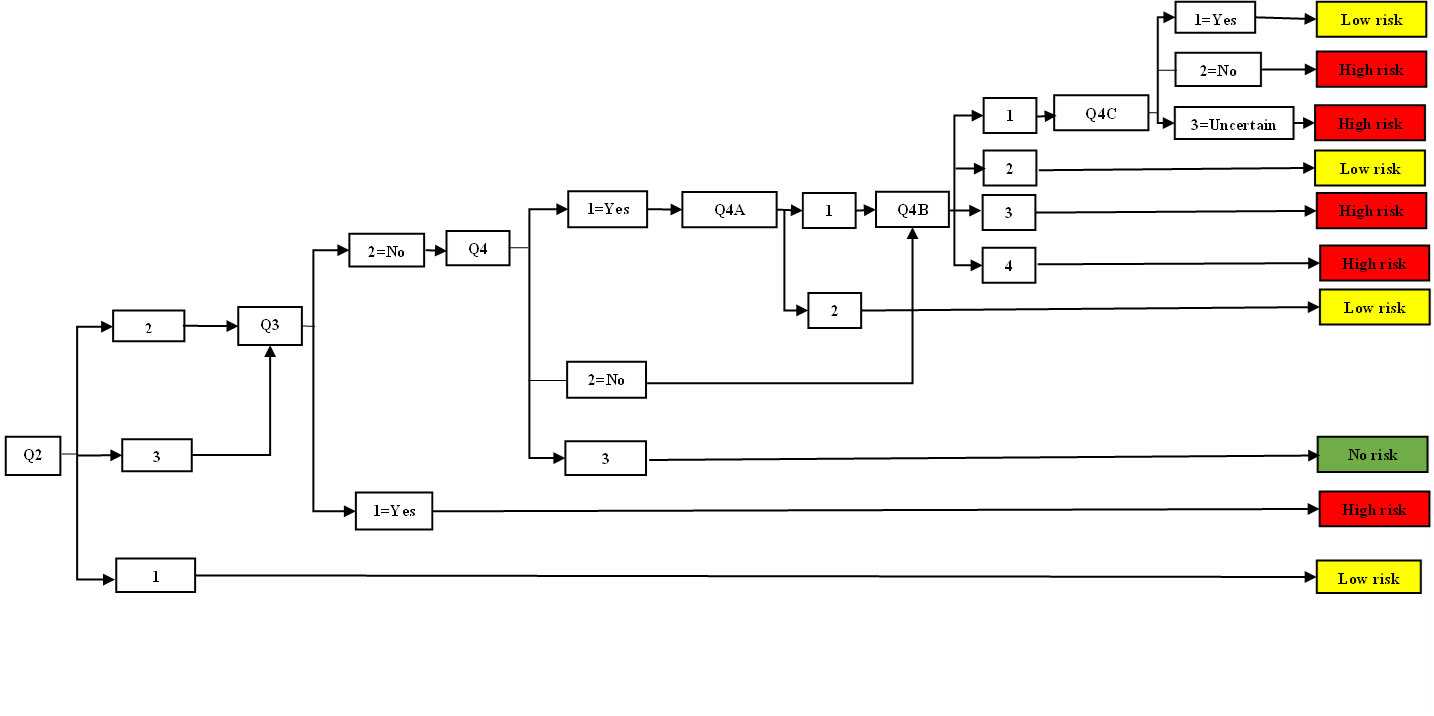


Figure 1. Flowchart of risk assessment in the HIVST-Chatbot
