# Supplementary material for: Evaluating an Innovative HIV Self-Testing Service With Web-Based, Real-Time Counseling Provided by an Artificial Intelligence Chatbot (HIVST-Chatbot) in Increasing HIV Self-Testing Use Among Chinese Men Who Have Sex With Men: Protocol for a Noninferiority Randomized Controlled Trial
Source: JMIR Res Protoc. 2023 Jun 30;12:e48447. doi: 10.2196/48447 (PMC10365592; doi:10.2196/48447)
Supplement: Multimedia Appendix 3 [file resprot_v12i1e48447_app3.pdf]

Developing and evaluating an innovative HIV self-testing service with online real-time instruction, pre-test and post-test counselling provided by a fully-automated artificial intelligence Chatbot (HIVST-Chatbot) (MSS349R)

Responses to reviewers' comments

1. It is stated that the need for this technology now is that the HIVST-OIC service is under pressure as a result of Covid related closures. However, Covid will pass so this need is only transient.

A: We have clarified that the need for the proposed technology is that the HIVST-OIC service is under pressure because of the manpower shortage along with the increasing demand of HIVST. Even before the COVID-19 outbreak, the demands of HIVST service among Chinese MSM has been increasing rapidly. The proportion of Chinese MSM who used HIVST had increased rapidly from 20.3% in 2012 to about 40% in 2018 (1-4). Similar increasing trend was also observed in Hong Kong (5). The HIVST-OIC makes HIVST more attractive to local MSM as it provides comprehensive support to the users. However, the HIVST-OIC requires intensive manpower to implement. For example, an administrator need to speed an hour to implement each episode of HIVST-OIC (including preparation, instruction of how to use HIVST kits, pre-test and post-test counseling). Therefore, the HIVST-OIC service was under pressure even before the COVID-19 outbreak. Although the non-governmental organization deployed more manpower to implement HIVST-OIC, the service capacity is still not able to satisfy the increasing demands of the community. We expect the demands of HIVST service will continue to grow even when COVID-19 pandemic goes to an end, just like the time before COVID-19.

References

[1] Han L, et al. HIV self-testing among online MSM in China: implications for expanding HIV testing among key populations. JAIDS, 2014; 67(2): 216-21

[2] Yan H, et al. Experiences and correlates of HIV self-testing among men who have sex with men in Jiangsu Province, China. AIDS and Behavior, 2015; 19(3): 485-91

[3] Ren XL, et al. Uptake of HIV self-testing among men who have sex with men in Beijing, China: a cross-sectional survey. Biomedical & Environmental Science, 2017: 30(6): 407-17

[4] Wei C, et al. Prevalence and correlates of point-of-sex HIV self-testing among HIV-negative men who have sex with men in China. Sexually Transmitted Diseases, 2018; 45(12): 812-22
[5] Centre for Health Protection. PRiSM --- HIV prevalence and risk behavioural survey of men who have sex with men in Hong Kong 2017. Available at: https://www.aids.gov.hk/english/surveillance/sur_report/prism2017e.pdf. Accessed on September 11, 2020

2. Page 11 states "80% of participants in the control group will take up HIVST" so the marginal effect of the Chatbot will only be on 10% overall. Is this significant to drive future uptake?
A: We have clarified that our assumption is that 80% of the participants in the control group (receiving HIVST-OIC) would take up HIVST, while at least 70% of those in the intervention group (receiving HIVST-Chatbot) would take up HIVST. The between-group difference would be not more than 10% (non-inferiority margin). If our hypothesis is proved, the effectiveness of HIVST-Chatbot in increasing HIVST uptake (at least 70%) would be no less than the HIVST-OIC, and be higher than that of some local HIV testing promotion program (e.g., 54.3% in a multimedia campaign launched by AIDS Concern in 2015) (1) and other HIVST promotion programs (32.7-54.7%) among Chinese MSM (2-4). Therefore, if the performance of HIV-Chatbot can meet our expectation, it would be as significant as the HIVST-OIC to drive HIVST uptake among local MSM and is cost-effective.

Reference

[1] Kwan NNM, et al. 'Get an early check --- Chrysanthemum tea': An outcome evaluation of a multimedia campaign promoting HIV testing among men who have sex with men in Hong Kong. HIV Medicine, 2018; 19(5): 347-354
[2] Tang W, et al. Crowdsourcing to expand HIV testing among men who have sex with men in China: a closed cohort stepped wedge cluster randomized controlled trial. PLoS Medicine, 2018; 15(8): e1002645
[3] Zhong F, et al. Acceptability and feasibility of a socio-entrepreneurship model to promote HIV self-testing and linkage to care among MSM. HIV Medicine, 2017; 18(5): 376-382

[4] Wang X, et al. Promoting oral HIV self-testing via the internet among men who have sex with men in China: a feasibility assessment. HIV Medicine, 2020; 21(5): 322-333

3. What about non-social media users?

A: We have clarified that one inclusion criterion is having internet access, as this is a prerequisite to receive HIVST-OIC or HIVST-Chatbot. The research team will facilitate participants to install WeChat/WhatsApp if needed and help them connect to the Chatbot. The internet access and smartphone ownership was very high among people in Hong Kong (97.3-99.8% and 95.9-99.3% among Hong Kong people aged 15-54 years) (1). One study reported the smartphone ownership was 93.6% among Chinese MSM in 2015 (2). Such figures are increasing overtime. Therefore, we expect very few MSM will be excluded because they do not have internet access. Given the increasing trend in internet access and smartphone ownership, inaccessibility would not be a barrier to receive HIVST-OIC or HIVST-Chatbot in future.

References

[1] Census and Statistics Department. Hong Kong Monthly Digest of Statistics: Usage of information technology and the Internet by Hong Kong residents, 2000 to 2016. Available at: https://www.statistics.gov.hk/pub/B71711FB2017XXXXB0100.pdf. Accessed on September 11, 2020

[2] Bien CH, et al. Gay apps for seeking sex partners in China: implications for MSM sexual health. AIDS and Behavior, 2015; 19(6), 941-946.

4. Ennui is not mentioned. Even AI driven responses will be come repetitive or off-topic after extended usage, how will this be avoided?

A: To address the potential issues related to ennui, we will build an evolving knowledge graph and our Chatbot will use a mixture of techniques so that a dialogue will not merely be rule-based, but it can also learn from a big pool of prior conversation pattern through machine learning.

1) We will build a knowledge graph that is evolving over time with increasing amount of prior conversations.

2) Our Chatbot will document prior conversations with the users. It will avoid giving a repetitive response to users' questions.
3) Our Chatbot will resolve from the recent conversation to create a plan of a suitable response. Relevant information will be retrieved from a big pool of prior conversation and used to prepare a reply. The Chatbot will select a persuasion template in accordance to the current stance of conversation and the perceived user characteristics. The pool of prior conversation pattern will grow bigger after extended usage and the variety of responses will increase accordingly. This approach is helpful to prevent repetitive or off-topic responses.

5. Future upkeep is not covered. Software development is often great but there is no mechanism to keep it updated and the Chatbot will eventually "die".
A: We agree with the reviewer that Chatbot maintenance is important. The research team will maintain the healthy state of the Chatbot using the following methods:
1) As mentioned in the response to comment #4, we will build a knowledge graph that is evolving overtime with increasing among of prior conversations. The Chatbot can learn from a big pool of prior conversation pattern through machine learning. This will allow the Chatbot to be increasingly relevant, helpful, and intelligent to meet users' expectation.
2) The interactions between the Chatbot and users will be reviewed monthly. The research team will identify sections of conversations that leads to users disconnecting the chat and find out whether there is anything in common between instances. In addition, the team will also look for other areas in which the Chatbot is underperforming. Improvement will be made to improve its performance.
3) A feedback loop will be incorporated into Chabot's interaction with the users. Users are invited to leave commentary before they complete the chat with the Chatbot. The research team will examine the feedback and improve the performance of the Chatbot.
