# Supplementary material for: Evaluating an Innovative HIV Self-Testing Service With Web-Based, Real-Time Counseling Provided by an Artificial Intelligence Chatbot (HIVST-Chatbot) in Increasing HIV Self-Testing Use Among Chinese Men Who Have Sex With Men: Protocol for a Noninferiority Randomized Controlled Trial
Source: JMIR Res Protoc. 2023 Jun 30;12:e48447. doi: 10.2196/48447 (PMC10365592; doi:10.2196/48447)
Supplement: Multimedia Appendix 2 [file resprot_v12i1e48447_app2.pdf]

愛滋病信託基金委員會
Council for the AIDS Trust Fund

愛滋病信託基金委員會秘書處
衛生署
衛生防護中心
香港九龍亞皆老街147C號四樓

Secretariat,
Council for the AIDS Trust Fund
Centre for Health Protection
Department of Health
4/F, 147C Argyle Street
Kowloon, Hong Kong

Our ref: ( ) in DH/ERPM/PMVD/P4-1/1(C)
Your ref:

22 December 2020

Centre for Health Behaviours Research,
JC School of Public Health and Primary Care,
The Chinese University of Hong Kong
Room 508, School of Public Health,
Prince of Wales Hospital,
Shatin, NT, Hong Kong
(Principal investigator: Prof. Zixin WANG)

Dear Prof WANG,

MSS 349 R: Developing and evaluating an innovative HIV self-testing service with online real-time instruction, pre-test and post-test counselling provided by a fully-automated artificial intelligence Chatbot (HIVST-Chatbot)

I am pleased to inform you that the Council for the AIDS Trust Fund (the Council) has approved the captioned AIDS Trust Fund application for a period of 2-year from May 2021 to April 2023. The detailed provisions for the approved project are set out at **Annex A**.

The grant for the first year will be released after we have received your written confirmation of acceptance of the approved grant and half of the second year's grant will be released at the beginning of the second year. You are required to submit a progress report within two months and an audited statement of accounts (including auditors' report) for the first year within six months after the commencement of the second year. Upon receipt of the report and subject to the satisfaction of the Council, the 40% of the second year's grant will be released in the middle of the second year.

The remaining 10% of the second year's grant will be released subject to the submission of a satisfactory final report within two months and an audited statement of accounts (including auditors' report) within six months of the completion of the project.

You should not accept the approved grant if you consider it is insufficient to achieve the objectives and indicators set out at **Annex B**.

Furthermore, you are required to make the following disclaimer on the articles (including webpage content, audio-visual materials, sound recordings, pictures and written materials) funded by AIDS Trust Fund: **"This HIV/AIDS prevention part of project / programme is sponsored by the AIDS Trust Fund. The content of this article represents the opinion of our organization only. It does not represent the position of the AIDS Trust Fund. AIDS Trust Fund is not responsible for any claims, demands or liabilities whatsoever arising from or in connection with the use of any information contained in this article or the participation of the sponsored project / programme."**

You should also note that all details of the publications related to this approved project (including journal name and date of publication) should be sent to the Council for information.

You should also note that re-allocation of funds among the approved items of expenditure and any modifications of the contents of the approved items (not involving additional funding) of an approved programme / project **are not allowed without the prior written approval** of the Council.

Please sign and return the attached "**Confirmation of Acceptance**" at **Annex C** if you accept all the conditions of grant. By signing the "Confirmation of Acceptance", you will be taken to have accepted the conditions of the grant as set out in the "**AIDS Trust Fund Medical and Support Services and Publicity and Public Education Application Guidelines**" and in Annexes A and B.

If the "Confirmation of Acceptance" is not signed and returned to us within 14 calendar days of this letter, the Secretariat will assume that you refuse to accept the grant and your application will be deemed withdrawn. Please quote the project reference: **MSS 349 R** in all future correspondence.

The comments from Sub-committee and Council members are extracted in **Annex D** for your reference.

Should you have any queries, please contact Dr. Silvia LAM at 2125 2181.

Yours sincerely,

(Dr. Ada LIN)
Secretary
Council for the AIDS Trust Fund

c.c. DH/ERPM/PMVD/P4-2/3/13

Annex D

Comments from Technical Reviewers, members of Medical and Support Services (MSS) Sub-Committee and Council for the AIDS Trust Fund are extracted as follows:

MSS 349 R: Developing and evaluating an innovative HIV self-testing service with online real-time instruction, pre-test and post-test counselling provided by a fully-automated artificial intelligence Chatbot (HIVST-Chatbot)

Technical reviewers

(a) Comments from Technical Reviewer A

1. This is a novel application to automate the encouragement of HIV self testing in MSM by using a Chatbot, an artificial intelligence trained automatic information resource, essentially replacing phone or face-to-face consultations so demanding less resource in the future. This type of health resource has appeared in a number of areas and the applicants have previous experience with this technology for smoking cessation. In some future time I assume AI driven responses will be much more widespread than today so application to HIV management is sure to occur. What I cannot judge however is how likely it is that the application will be successful right now.

2. The application is confident and the numbers seem to be sufficient for a statistically significant outcome. I did not find any obvious problems with what is proposed as it follows similar developments in other areas, no crucial new technicology needs to be developed. Even the responses the Chatbot will use will be based on the existing HIVST-OIC service so there is little reason it cannot be developed. My main concerns are over take-up and the level of overall benefit as follows.

- It is stated that the need for this technicology now is that the HIVST-OIC service is under pressure as a result of COVID related closures. However, COVID will pass so this need is only transient.
- Page 11 states "80% of participants in the control group will take up HIVST" so the marginal effect of the Chatbot will only be on 10% overall. Is this significant to drive future uptake?
- What about non-social media users?
- Ennui is not mentioned. Even AI driven responses will be come repetitive or off-topic after extended usage, how will this be avoided?
- Future upkeep is not covered. Software development is often great but there is no mechanism to keep it updated and the Chatbot will eventually "die".

3. I scored the application quite highly as it is technically feasible to develop and test and of course better self testing in the MSM population would be beneficial. But I think these longer term issues should be considered if it is funded.

(b) Comments from Technical Reviewer B

4. The study may support remote HIV testing and counselling in settings where face-to-face contact is not possible such as the current COVID-19 situation.

5. The use of two methods of HIV testing (oral and blood based), introduces a possible source of bias as this test format itself may influence participants response to counseling. The Chatbot will provide different health education information depending on their initial risk profile. It would seem important to provide all participants with the same HIV prevention information as individuals risk behaviors may change over time.

6. The resources proposed to support the development of the fully-automated Chatbot, which include building up cloud server and computing infrastructure, appear excessive and beyond the scope of the research aims.

Recommendation of MSS Sub-Committee

7. With the advancement in technology and use of AI in various fields nowadays, the MSS Sub-Committee agreed with the comments from technical reviewer A that the application of AI driven responses to HIV management would happen in some future time. The findings of this study on the efficacy of AI Chatbot in self-testing services are important, especially during the COVID-19 pandemic when the traditional facility-based HIV/AIDS services had been disrupted. In addition, the materials developed will be available to all NGOs for prevention and non-commercial purposes. Taking the above into account, the MSS Sub-Committee recommended to support the study.

Council for the ATF

8. The Council **endorsed** the recommendation of MSS Sub-Committee to provide funding support.
